# Nondysraphic Intradural Spinal Lipoma: A Case Report and Review of Pathogenesis

**DOI:** 10.7759/cureus.66481

**Published:** 2024-08-08

**Authors:** Shehzaib Raees, Maigrie McDougal, Abigail Zaratan, Jason Chang, Forshing Lui

**Affiliations:** 1 Clinical Sciences, California Northstate University College of Medicine, Elk Grove, USA; 2 Neurology, Kaiser Permanente South Sacramento Medical Center, Sacramento, USA

**Keywords:** neural tube defects, intradural spinal lipoma, nondysraphic lipoma, thoracic lipoma, spinal lipoma, lipoma

## Abstract

Intradural spinal lipomas are rare benign lesions typically located in the lumbosacral region and associated with spinal dysraphism in children. When unassociated with spinal dysraphism, they are most often diagnosed in young children or adolescents following the emergence of neurological symptoms. In their most rare form, intradural spinal lipomas may be found in adults without spinal dysraphism. Here, we present a case of a 42-year-old female with an intradural spinal lipoma without dysraphism at the T10-T11 level, demonstrating the diagnostic challenge of atypical lipomas and the importance of timely assessment and management. We also reviewed the embryopathogenesis of the different types of intradural spinal lipomas and the importance of surgical interventional planning and approach.

## Introduction

Intradural spinal lipomas are rare benign lesions that consist of cells similar to normal adipose tissue and are typically present in the lumbosacral region of young patients with spinal dysraphism [1]. Spinal lipomas without dysraphism are exceedingly rare; most documented cases are in children and adolescents who have abrupt, severe neurological defects [2]. We report a case of a 42-year-old female with a 20-year history of back pain that evolved into more significant neurological deficits. The patient was diagnosed with an intradural spinal lipoma without dysraphism at the T10-11 level. This case is unique given the patient’s older age at the onset of neurological symptoms and the rarity of spinal lipomas without dysraphism [3]. We believe that this case report will allow clinicians to understand the atypical presentation of spinal lipomas, as well as to understand several factors that may be implicated in the pathogenesis of nondysraphic spinal lipomas later in life.

## Case presentation

The patient is a 42-year-old female former gymnast with a past medical history of severe obesity with a 44-pound weight gain in the past 10 years. She presented to her primary care doctor on 01/2022 with chronic, periodically worsening lower back pain for the previous 20 years and lower extremity weakness for the past year. She had experienced numbness and tingling that originated in her left lower limb and eventually affected the right lower extremity. This numbness radiated from the bottom of the foot to the knee and wrapped around the legs without a particular dermatomal distribution. This sensation was triggered by position and movement when she stretched her arms above her head, as well as when she transitioned from sitting to standing. She had a subsequent episode of tingling in her right foot that radiated up to the knee when walking up the stairs followed by knee instability, resulting in a fall. She experienced weakness starting at the hip flexors that extended to the entirety of her legs bilaterally. The patient had no complaints of urinary incontinence or incomplete emptying. The patient’s family history was notable for diabetes and breast cancer on her paternal side.

On physical exam, the patient was well-appearing and in no acute distress, alert and oriented with normal mentation and language. Vital signs were within normal range. Her weight was 253 pounds, and her height was 64 inches with a BMI of 43.4 kg/m^2^ (Class 3 obesity). A spinal exam revealed no tenderness with a full range of motion, as well as no midline tufts of hair or midline spinal abnormalities. Cranial nerves II-XII were normal. Left hip flexion was decreased (4/5 motor strength), but all other strength exams were 5/5. The patient exhibited a positive left Babinski sign and hyperreflexia of both ankle jerks with unsustained ankle clonus (left > right). She had a patchy reduction of pinprick and temperature sensation in the lateral thighs and legs bilaterally without a dermatomal distribution. Proprioception and coordination were intact. Gait exam was normal, including tandem walking, except for subtle circumduction of the left hip.

**Figure 1:**
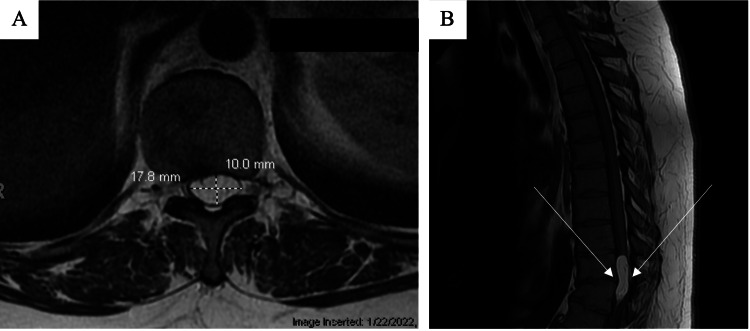
Pre-operative MRI of the spine A) T1 sagittal view MRI of L-spine showed an intradural 1.1 x 1.4 x 4.0 cm mass at the distal thoracic cord level at T10 and T11. B) T2 axial view showed hyperintense lesion consistent with a diagnosis of thoracic lipoma.

An MRI of her spine was performed. It showed an intradural mass (4.0 cm x 1.4 cm x 1.1 cm) at the distal thoracic spinal cord at T10-T11 levels. The mass was hyperintense in both T1- and T2-weighted sequences, consistent with an intradural spinal lipoma with a rightward displacement of the spinal cord due to a significant mass effect (Figure 1). Imaging also showed T11 anterior compression fracture and multilevel degenerative disc disease with low-grade spinal canal or foramen stenosis.

**Figure 2:**
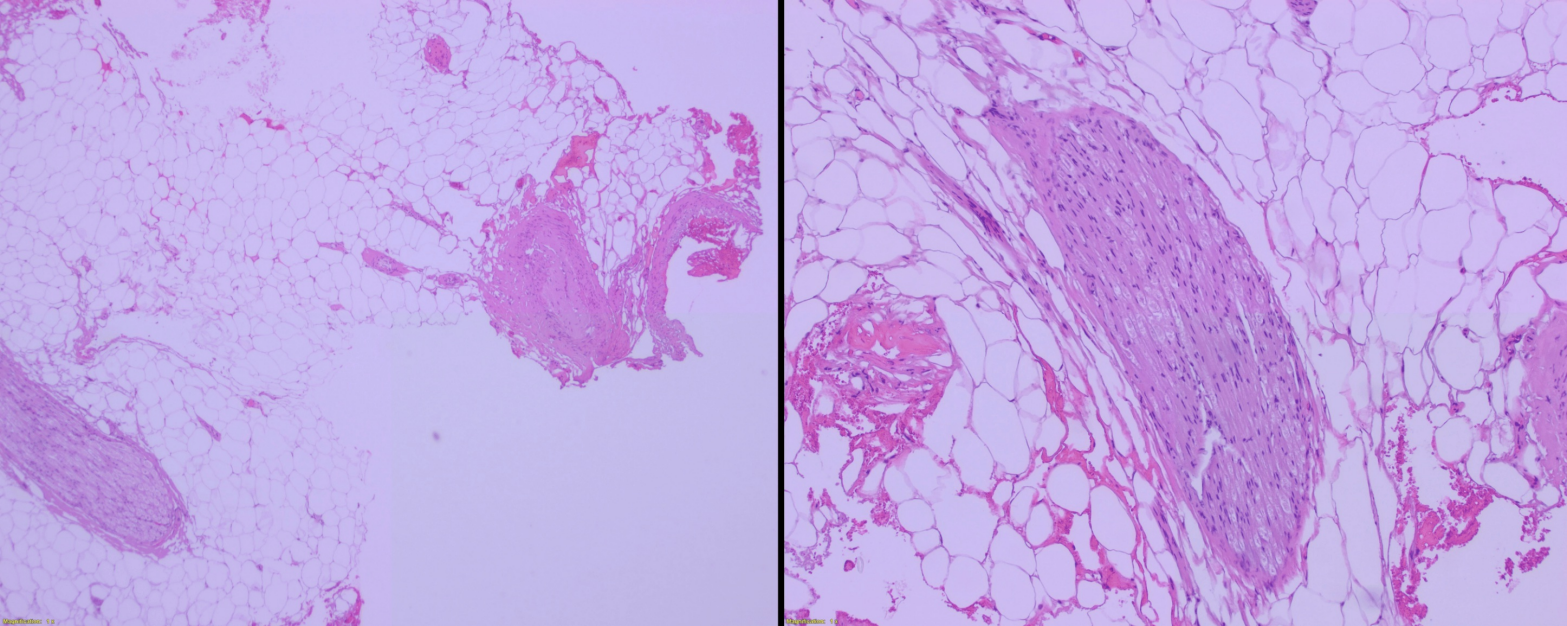
Histopathology slides of intradural spinal lipoma Mature fibroadipose tissue mixed with fragments of blood vessels and peripheral nerves typical of a transitional type cord lipoma.

Given the patient’s history, physical exam findings, and imaging studies, she was diagnosed with spinal cord compression due to intradural spinal lipoma at T10-T11. The patient subsequently underwent T10-T11 laminectomy for intradural lipomatous mass exploration with debulking and untethering of the spinal cord. Intraoperatively, a fatty lesion on the left dorsal aspect of T10-11 extending towards the filum terminale was visualized, grossly compatible with a transitional lipoma that was adherent to the spinal cord with no clear margins between the spinal cord and nerve roots. As a result, the surgeon did not attempt a radical resection or complete removal of the fatty tissue but rather proceeded with careful debulking of the lipoma. The histopathology showed mature fibroadipose tissue mixed with fragments of blood vessels and peripheral nerves representative of a transitional type cord lipoma (Figure 2).

**Figure 3:**
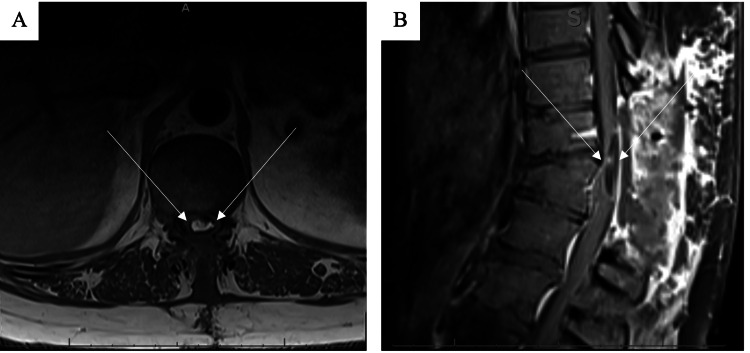
Post-operative MRI of the spine A) Post-operative T1 sagittal MRI of the thoracic cord showed a residual 3.8 cm x 1.1 cm x 0.6 cm lesion at the level of T11. B) Post-operative T1 axial MRI shows moderate to severe central canal stenosis and mass effect, improved from prior examination.

Post-operative imaging showed a residual lesion (3.8 cm x 1.1 cm x 0.6 cm) with moderate to severe central canal stenosis and mass effect on the thoracic cord displacing it to the right (Figure 3). However, both mass effect and central canal stenosis were improved from the initial examination and MRI about six months prior. Post-operative EMG/NC was normal with no lumbosacral radiculopathy or peroneal neuropathy.

Upon follow-up on 11/2022, the patient’s lower extremity weakness improved, except for 4+/5 left hip flexion. Her intermittent right leg weakness and radiating sensory symptoms also resolved. However, the patient had persistent patchy numbness and rare shooting pain in the L2-L3 dermatome, in addition to reduced temperature/pinprick sensation in the right lower extremity, and 3-4 beats of left ankle clonus. The Babinski sign was also present on the left. These signs likely represented persistent upper motor neuron damage despite the successful debulking procedure.

## Discussion

Spinal lipomas encompass a large collection of benign malformations ranging in pathophysiology and clinical presentation. While most occur in the lumbosacral region, they can occur at all levels of the spine and present differently based on the level affected [4].

Intradural spinal lipomas are extremely rare; they make up approximately 1% of all spinal tumors and are normally associated with spinal dysraphism [2]. Spinal lipomas unassociated with spinal dysraphism are typically diagnosed in children and young adults following a symptomatic event or progression in symptoms [1]. Depending on the location of the lesion, symptoms can include spastic weakness in the extremities, sensory changes, or gait difficulties [1]. In general, spinal lipomas without spinal dysraphism are exceedingly rare with an approximate incidence of 0.45-0.6% of all spinal tumors [3]. In the literature, most cases of nondysraphic spinal lipomas are described in children or young adults under 20 years of age and occur in the lumbosacral region [2]. Nondysraphic spinal lipomas of the thoracic and cervical levels are more uncommon [2]. The characteristic early appearance of symptoms in nondysraphic spinal lipoma could be due to the limited space for expansion prior to impinging on other nervous system structures [1]. Due to the nature of spinal lipomas, it is possible that this condition may be underdiagnosed in the general population [4].

The pathophysiology of spinal lipomas differs between children and older adults, as the underlying causes and manifestations of the condition vary between these age groups. Spinal lipomas in children are associated with developmental abnormalities in the spinal cord. These congenital spinal lipomas can occur because of failure of the neural tube to close (primary neurulation) during normal embryonic development [4]. The pathology of intradural spinal lipomas in children and adults consists of mature adipose tissues mixed with mature fibrous tissues, blood vessels, and nerves. These lipomas are hamartomatous rather than real neoplastic benign lesions elsewhere in the body. The adipose tissue will behave similarly to other body fat in the patients. As the spinal cord grows, fatty tissue can accumulate within the spinal cord due to these defects, which then leads to a lipoma that is typically present at birth [4]. In children, spinal lipomas are more likely to cause symptoms that manifest early in life such as urinary or bowel incontinence, difficulty walking, and scoliosis [4].

In our case, the patient presented with an intradural spinal lipoma at the level of T10-11. Pathology was compatible with a transitional type lipoma, a combination of caudal and dorsal types of lipoma, which has a large oblique placode-lipoma interface [4]. Typically, the rostral end of a transitional lipoma will resemble a dorsal lipoma, and as the lipoma continues distally, it will rotate ventro-caudally, making a slanted line eventually involving the entire conus [5]. Since this type of lipoma is frequently asymmetrical, it is often difficult to detether [4]. The embryological origin of spinal cord lipomas remains a matter of speculation; however, there are several prominent theories regarding possible errors in neurulation [4,5]. In lipomas typically associated with dysraphism, including dorsal lipomas, the primary error is thought to be premature ectodermal separation during primary neurulation, leading to the migration of cutaneous ectodermal and mesenchymal tissues into the open neural tube [5]. However, the involvement of the filum in transitional-type lipomas indicates a further error in secondary neurulation. Thus, the overarching hypothesis is that transitional lipomas result from premature ectodermal disjunction during primary neurulation and abnormal condensation during secondary neurulation [5]. This is reflected in the slanted shape of most transitional lipomas, as well as their irregular fusion line [5]. Understanding the pathology of these different types of spinal lipomas will help the planning of a surgical approach to the treatment of these patients.

Intradural spinal lipomas are typically present at birth but may be asymptomatic until the second to third decades of life. The most common symptoms are spinal pain, dysesthetic sensory change, gait difficulties, weakness, and incontinence [6], typically in a progressively worsening, relapsing-remitting pattern [3]. The mechanism for the presentation of these symptoms has been widely debated, and there are several theories attempting to describe why they appear [6]. The first and most widely accepted is that spinal lipomas cause increasing mass effects over time due to their progressive growth. This increased mass effect causes a gradual compression and/or displacement of the spinal cord which may in turn cause neurological signs and symptoms. The mass effect from the patient’s transitional type lipoma manifested as numbness in her anterior lower extremities when going from the sitting to standing position, indicating nerve root compression explained by central stenosis [7]. Central stenosis may cause neurogenic claudication due to impaired epidural venous blood flow to the cauda equina and increased vascular pressure [8]. It is typically asymptomatic at rest but can cause symptoms when the patient is walking or standing because the spinal canal naturally narrows when the lumbar spine is extended, further compressing the spinal cord [8]. This results in many patients maintaining a stooped posture in order to prevent compression from occurring [7].

Another potential cause of symptoms is tethering of the spinal cord, which results in hypoxia and subsequent ischemia [9]. This spinal cord ischemia impairs oxidative metabolism and reduces electrical activity, which can result in impaired sensation, loss of motor strength, and other upper motor neuron signs [9]. This theory also explains why repeated flexion and extension movements of the spine can worsen symptoms, as blood flow to the spinal cord cannot keep up with the increased metabolic activity of the nerve roots in the lower extremities during walking or standing [7,10]. Thus, neural ischemia and defective nerve conduction may occur, particularly with lumbar spinal stenosis [8]. The patient exhibited symptoms of decreased lower extremity muscle strength, impaired balance, numbness, and tingling with physical examination signs showing upper motor neuron damage with a positive Babinski reflex, clonus, and hyperreflexia, all of which are suggestive of thoracic spinal stenosis [11]. Surgical resection and debulking of the lipoma in this patient’s case improved the central canal stenosis and mass effect.

It is fair to assume that both theories may explain the onset and subsequent worsening of symptoms when examining our patient’s weight changes as it increased from approximately 207 pounds on 4/30/2010 to 251 pounds on 2/2/2022, representing a 44-pound increase in weight.

Previous case reports indicated a positive correlation between spinal lipoma size and overall body fat changes, with some literature indicating that this correlation may not be particularly strong in all patients [12]. The progressive and significant weight gain of 44 pounds in 10 years in our patient can at least in part be associated with concomitant enlargement of the lipoma as part of hypertrophy of adipose tissues. The growth of the lipoma led to more symptoms in our patient. Most patients tend to show symptoms for more than two years prior to the first hospital visit, with patients that have cervical spinal lipomas presenting almost 10 years after the onset of symptoms [13]. Our patient followed a similar course, with constant and occasionally worsening back pain for 20 years with additional neurological signs in the past year.

While the underlying causes and manifestations of spinal lipomas can differ between older adults and children, the treatment for both conditions typically involves debulking surgery to reduce the size of the lipoma and provide symptomatic relief [13]. In most cases, however, complete removal of the lipoma is unwarranted and difficult due to the ill-defined fusion line between the transitional lipoma and spinal cord, poorly defined caudal demarcations, and the larger surface area of fat coverage [5]. Despite being the preferred option, partial spinal lipoma resection recurrence rates tend to be between 46% and 52% depending on the population, which may challenge the efficacy of the current guidelines on debulking procedures [5]. The recurrence after partial resection of the lipoma is hypothesized to occur because of the residual fat and its potential to produce adhesions for retethering, which later manifest as neurological deterioration and other motor or sensory changes [5,14].

## Conclusions

Intradural spinal lipomas are rare benign lesions and may be underdiagnosed, especially in adult populations. Due to the rarity of the condition, the most appropriate surgical management has been subject to debate. The patient in this case went through a partial resection and showed significant improvement following the laminectomy and debulking, with resolution of the right lower extremity weakness and radiating pain.

This case is unique in regard to the onset of symptoms associated with intradural spinal lipomas, given the patient’s older age and prolonged asymptomatic phase. Understanding the embryological development and pathophysiology of spinal lipomas will help improve diagnostics and treatment as it provides a deeper insight into symptom relief and the risks and benefits of different surgical approaches.
